# A multicenter study on occult lymph node metastases in sinonasal malignancies

**DOI:** 10.1038/s41598-026-47890-z

**Published:** 2026-05-24

**Authors:** Christina Sauter, Nina Wenda, Miray-Su Yilmaz Topçuoğlu, Maximilian Linxweiler, Noran Elawany, Charlotte Voigt, Marc Scheithauer, Patrick J. Schuler, Johannes Zenk, Thomas K. Hoffmann, Johannes Doescher

**Affiliations:** 1 Department of Otolaryngology, Augsburg University Hospital, University Hospital Augsburg, Sauerbruchstr. 6, 86179 Augsburg, Germany; 2 Department of Otolaryngology, Head and Neck Surgery, Helios HSK, Wiesbaden, Germany; 3 Department of Otorhinolaryngology- Head and Neck Surgery, University Hospital Heidelberg, Heidelberg, Germany; 4 Department of Otorhinolaryngology, Head and Neck Surgery, Saarland University Medical Center, Homburg, Germany; 5 Department of Otorhinolaryngology, Head and Neck Surgery, University Medical Center Ulm, Ulm, Germany

**Keywords:** Sinonasal malignancy, Sinonasal cancer, Occult lymph node metastasis, Elective neck dissection, Head and neck cancer, Patterns of failure, Cancer, Oncology

## Abstract

Surgical interventions for head and neck carcinoma patients with clinically node-negative neck encompass either elective neck dissection (ND) during primary tumor excision or a wait-and-scan approach. For the rare entity of sinonasal malignancies, no standard guideline exists. This study aims to assess the prevalence of occult lymph node metastasis and evaluate the impact of elective ND on recurrence and survival. This retrospective multicenter study included patients with histologically confirmed sinonasal malignancy between 2012 and 2022. Patient-, disease-, and therapy-specific factors were systematically collected and analyzed. The therapeutic approach was based on recommendations from an interdisciplinary head and neck tumor board. A total of 438 patients with a mean age of 62 years was included. Occult metastases were observed in 8.0% of all cN0 patients assessed by objective imaging. Elective ND was performed in 25% of patients. Histologic subtype of squamous cell carcinoma (SCC) was the only pretherapeutic risk factor identified (OR 6.10; *p* = 0.019). Five-year disease-free survival (DFS) was significantly reduced in patients with occult lymph node metastasis (*p* < 0.001). This multicenter study demonstrates a comparatively low risk of occult lymph node metastases in sinonasal malignancies, but significantly reduced DFS. The low rate of occult metastases questions the necessity of systematic elective ND. The indication for elective ND should be critically assessed depending on histology.

**Electronic supplementary material:**

The online version of this article (10.1038/s41598-026-47890-z) contains supplementary material, which is available to authorized users.

## Introduction

Malignant tumors of the nasal cavity and paranasal sinuses account for approximately 3–5% of all malignant neoplasms of the upper respiratory tract. Representing less than 1% of all malignancies, with an incidence of 0.5–1 new diagnoses per 100,000 inhabitants, they are overall rare^1,2^. Histologically, they constitute a heterogenous group: Squamous cell carcinoma (SCC) is the most common (48.7%), followed by adenocarcinoma and other entities ^3^. The standard treatment for SCC of the nasal cavity and sinuses involves the surgical excision of the primary, preferably through an endoscopic approach^4,5^. The latter approach is considered less invasive and has been shown to facilitate more expeditious rehabilitation and the timely initiation of adjuvant therapy^6^.

In general, the presence of regional metastases of the cervical lymph nodes is uncommon at the time of diagnosis. The incidence of such metastases is documented to be approximately 10% in a series by Peck et al.^7^. Furthermore, up to 33% of patients experience lymph node metastases during subsequent follow-up^7,8^. The overall survival is lower for patients with positive nodal status (N1: hazard ratio (HR) of 1.56; N2/3: HR of 1.6) compared to patients with N0 status^9^. Patients diagnosed with SCC exhibit a greater likelihood of developing lymph node metastases compared to other entities, displaying an average of 12.6% for both initial metastases and recurrences^7,10–16^.

In the event of radiological and clinical suspicion of lymph node metastases in the neck area, neck dissection (ND) constitutes an integral component of the therapeutic regimen, similar to other tumors of the head and neck region^4,5^. Surgical interventions for patients with clinically node-negative neck encompass either elective ND at the time of the excision of the primary, or a wait-and-scan approach aimed at early detection of lymph node metastasis and secondary therapeutic ND. A paucity of consensus exists with regard to the standardization of the procedure. In a meta-analysis by Galloni et al. a significantly improved locoregional control rate after elective ND of an N0 neck has been described^17^. This finding was corroborated in another meta-analysis of sinonasal undifferentiated carcinomas (SNUC)^18^. Conversely, other authors do not advocate for elective ND in the absence of lymph node metastases, even in cases of advanced T-stage^13,19^. The existent literature contains a limited amount of data regarding the detection of occult lymph node metastases subsequent to elective ND; however, a rate of 12.5% was described in a recently published systematic review^20^. A number of treatment concepts have been developed, including sentinel lymph node biopsy. In this procedure, the sentinel lymph node—which is affected first in the lymphatic drainage area of a primary tumor in the case of lymphogenic metastasis—is removed for subsequent histological examination. A pilot study using this method identified sentinel lymph nodes in four of six patients, and an occult lymph node metastasis in one of these cases^21^.

The objective of this study is to ascertain the prevalence of positive pathological lymph node findings either following ND for sinonasal malignancy or as isolated lymph node recurrence in the follow-up. Herewith, we want to determine the effectiveness of elective ND in reducing recurrences and enhancing survival rates.

## Materials and methods

### Patient cohort

This study was approved by the institutional review board of the Ludwig Maximilians University of Munich, Germany (Munich, Germany; Project number 22–0963). Owing to the retrospective study design and the analysis of pseudonymized clinical data, the requirement for informed consent was waived by the ethics committee. All experiments were performed in accordance with relevant guidelines and regulations.

This retrospective multicenter study included patients with a histologically confirmed diagnosis of sinonasal malignancy and clinical N0 status between 2012 and 2022. All patients were recruited of one of the following Ear, Nose and Throat, Head and Neck surgery departments in Germany: University Hospital of Augsburg, University Hospital of Heidelberg, University Hospital of Saarland, University Hospital of Ulm, Dr. Horst-Schmidt Hospitals Wiesbaden. Patients with lymphoma of the sinonasal tract were excluded. All data were collected retrospectively from the patient files of the respective clinic and processed pseudonymously. Patient-, disease- and therapy-specific factors were collected and correlated with each other.

The radiological findings were critically evaluated with regard to the suspicion of lymph node metastases. The therapy was standardized in each patient and followed the decisions of an interdisciplinary head and neck tumor conference. Occult lymph node metastases were defined as the occurrence of simultaneous lymph node metastasis subsequent to elective ND in the cN0 neck (cN0/pN +), or as a distinct manifestation of isolated lymph node recurrence in conjunction with a prior clinically node-negative neck without ND (rT0rN +).

According to our theory, isolated lymph node recurrence is most likely attributable to a previously existing occult lymph node metastasis. It is important to note that this definition does not encompass combined locoregional recurrences.

### Statistical analysis

Statistical analysis was performed with the Statistical Package for Social Sciences software, version 29.0.2 (SPSS, Chicago, IL). Diagrams were created using GraphPad Prism version 10.1.1 (GraphPad Software, Boston, MA). Correlation analysis was conducted using the chi-square test and Fisher’s exact test for categorical or nominal variables when investigating differences. For evaluating risk factors for occult metastasis, we utilized binary and multinomial logistic regression analysis, followed by a multivariate analysis that incorporated factors with a significance of *p* < 0.2 in the univariate analysis. In addition, disease-free (DFS) and overall survival (OS) in patients with and without occult metastasis were estimated using the Kaplan–Meier method. We utilized the log-rank test to compare survival outcomes between the two groups. DFS was defined as the time elapsed from the day of surgery to locoregional or lymph node/distant metastatic recurrence, or death and was censored on the last day the patient was alive without any evidence of recurrence. Additionally, in cases involving occult lymph node metastases, an adjusted disease-free survival was calculated, beginning after therapeutic neck dissection of lymph node metastasis. OS was defined as the time from the day of resection to death from any cause and was censored at the last day when the patient was alive. To circumvent the possibility of confounders, significant results of the log-rank test (*p* < 0.2) underwent a multivariate analysis employing the Cox regression model. A two-sided p value of < 0.05 was considered statistically significant.

## Results

### Patients

A total of 438 patients diagnosed with sinonasal malignancy were treated at one of the aforementioned medical centers between 2012 and 2022. The median follow-up in this population was 30 months (range, 0.13–245). The most prevalent histologic subtype was SCC (55.3%), followed by adenocarcinoma (16.4%). The tumors were most frequently located in the nasal cavity (53.4%). The tumor size in our population was well balanced (T3-4: 49.1% vs. T1-2: 42.5%). The whole cohort had no clinical evidence of cervical lymph node metastasis at the time of diagnosis. The cervical lymph node status was evaluated through various imaging modalities, including computed tomography (CT) (65.2%) and magnetic resonance imaging (MRI) (11.6%) with or without ultrasound of the neck. A further 3.6% of patients underwent both CT and MRI scans, while 19.6% solely underwent ultrasound of the neck. Occult metastases were apparent in 35 patients (8.0%) and were characterized as the occurrence of simultaneous lymph node metastasis subsequent to elective ND in the cN0 neck (cN0/pN +), or as a distinct manifestation of isolated lymph node recurrence in conjunction with a prior cN0 neck without ND (rT0rN +). Patients diagnosed with SCC exhibited occult lymph node metastases in 10.3% of cases, while those diagnosed with neuroendocrine carcinoma (NEC) demonstrated an occurrence of 22.2% and those with SNUC in 10.5% (Table 1).

**Table 1:** Patient characteristics.

Characteristic		All patients (*n* = 438)	
Mean age (range, years)		62 (21–96)	
Sex			
Male		280 (63.9)	
Female		158 (36.1)	
Site of primary tumor			
Nasal cavity		234 (53.4)	
Paranasal sinuses		142 (32.4)	
Nose cavity and paranasal sinuses		62 (14.2)	
Morphologic subgroup		total	Occult lymph node metastasis*
Squamous cell carcinoma		242 (55.3)	25 (10.3)
Adenocarcinoma		72 (16.4)	3 (4.2)
Adenoidcystic carcinoma		18 (4.1)	0
Melanoma		33 (7.5)	0
Esthesioneuroblastoma		16 (3.7)	0
SNUC		19 (4.3)	2 (10.5)
Sarcoma		12 (2.7)	0
Neuroendocrine carcinoma		18 (4.1)	4 (22.2)
Other		8 (1.8)	1 (12.5)
Occult lymph node metastasis			
Yes		35 (8.0)	
	cN0/pN +	8 (22.9%)**	
	rT0rN +	27 (77.1%)**	
No		403 (92.0)	
Tumor stage			
cT1-2		186 (42.5)	
cT3-4		215 (49.1)	
cTx		37 (8.4)	

*percentage in relation to tumor entity.

**percentage out of all occult lymph node metastases.

*[SNUC* = *sinonasal undifferentiated carcinoma]*

### Therapy

The treatment of the primary was executed as either an open or endoscopic procedure (open: n = 179, 40.9%; endoscopic: n = 200, 45.7%). A total of 21 patients (4.8%) underwent a combination of open and endoscopic surgery. In 2.9% of patients, no statement could be made about the type of surgical treatment. Twenty-five patients (5.7%) received primary chemoradiotherapy in curative intent. Chemoradiotherapy was performed on 57 patients (13.0%) and radiotherapy on 187 patients (42.7%) as an adjuvant treatment, respectively. Elective ND was performed in 109 (24.9%) patients, with a majority of selective ND (69.6%). The mean number of lymph nodes removed during elective ND was 23 (95% confidence interval [CI], 19.9 to 26.9). Diagnostic lymph node removal was only conducted in eight patients (1.8%). Of these, four had a positive lymph node status and subsequently underwent therapeutic ND.

### Risk for occult lymph node metastasis

In summary, 35 patients (8.0%) exhibited occult lymph node metastases. Of these, eight (22.9%) presented after undergoing elective ND for a cN0 neck (cN0/pN +). The remaining 27 patients (77.1%) exhibited isolated lymph node metastasis subsequent to the completion of treatment without prior ND (rT0rN +). The median time to lymph node metastasis was nine months (95% CI, 3.2 to 15.2).

The majority of patients (26 out of 35) with occult metastasis exhibited a solitary lymph node metastasis, designated as N1. Three patients were found to have singular lymph node metastasis between 3 and 6cm on the ipsilateral side (N2a). About nine percent each had multiple ipsilateral lymph node metastases smaller than 6 cm (N2b; n = 3) and bilateral lymph node metastases (N2c; n = 3). Lymph nodes greater than 6 cm or extranodal extension, leading to N3 status, were not observed in any of the patients with occult lymphatic metastasis.

We conducted univariate and multivariate analyses to identify risk factors for occult metastasis in the cN0 neck. In the univariate logistic regression analysis, only histology emerged as prognostic indicator for occult metastasis. Specifically, SCC (Odds ratio [OR], 3.303; 95% CI, 1.33 to 8.23; *p* = 0.010) and NEC (OR, 8.190; 95% CI, 2.07 to 32.47; *p* = 0.003) were identified as significant risk factors. R0-status (OR, 0.333; 95% CI, 0.11 to 1.01; *p* = 0.052) revealed a lower risk for occult metastasis, although not statistically significant. Subsequently, the multivariate analysis only revealed SCC as an independent risk factor (*p* = 0.019). Detailed results of both the univariate and multivariate analyses can be found in Table 2.

**Table 2:** Univariate and multivariate analysis of the risk factors for occult lymph node metastasis*.*

		Univariate analysis			Multivariate analysis		
		OR	95% CI	p-value	OR	95% CI	p-value
Sex	male	0.951	0.47–1.95	0.891			
	female						
cT	cT 1–2	0.971	0.48–1.96	0.934			
	cT 3–4						
Grading	G1	0.000	–	0.998			
	G2-3						
Histology	**SCC**	**3.303**	**1.33–8.23**	**0.010**	**6.096**	**1.35–27.59**	**0.019**
	**NEC**	**8.190**	**2.07–32.47**	**0.003**	6.417	0.54–76.76	0.142
	other						
R status	R0	0.333	0.11–1.01	0.052	0.502	0.16–1.62	0.250
	R +						
Age	≤ 50	0.360	0.30–1.88	0.644			
	> 50						

[*NEC* = *neuroendocrine carcinoma; SCC* = *squamous cell carcinoma].*

Significant values are in [bold].

In the next step, we analyzed the distribution of occult metastasis while considering clinical T stage and tumor localization. Occult lymph node metastases occurred with similar frequency in tumor stages cT1-2 (n = 16; 8.6%) and cT3-4 (n = 18; 8.4%) (p = 0.462).

Occult metastases were found particularly in carcinomas of the main nasal cavity (n = 23; 65.7%). While 89.9% of these cases occurred in tumor stages T1-2, in the paranasal sinuses, they occurred exclusively in advanced tumor stages T3-4 (n = 7; 20%). In the context of combined tumors, the observed figure was 14.3% (n = 5).

### Overall survival

During the observation period, 81 patients (18.5%) died. Of these patients, approximately one third (n = 25; 30.9%) died as a direct consequence of the tumor or its therapy. A proportion of 42.7% died of a cause other than the tumor disease. For the remaining patients, no cause of death could be determined in retrospect.

There were 67 deaths (20.4%) in patients without ND, with 31.3% being tumor-related, and 14 deaths (12.8%) in the elective surgery group (28.6% tumor-related).

The five-year OS rates showed no significant difference, but a clear tendency towards elective ND [81.9% (95% CI, 84.2 to 79.6)] compared to patients without ND [73.9% (95% CI, 66.6 to 81.2)] (*p* = 0.145).

**Fig. 1:**
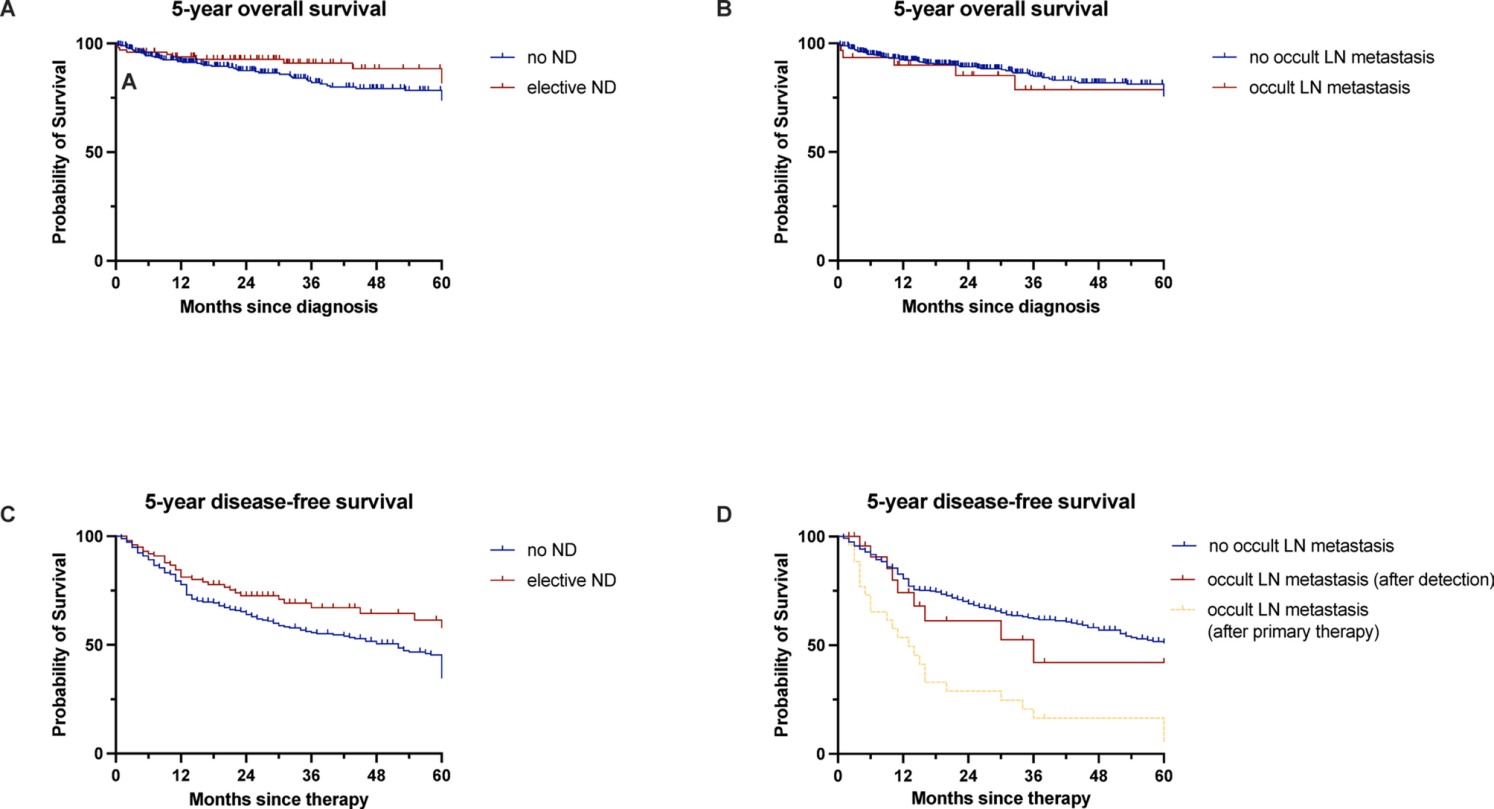
Five-year overall survival (**A–B**) and five-year disease-free survival (**C–D**) in patients depending on neck dissection and occult lymph node metastasis. [LN = lymph node; ND = neck dissection].

A percentage of 25.7% (n = 9) patients with occult lymph node metastases died, leading to a five-year OS of 78.7% (95% CI, 70.8 to 86.6), and 75.8% (95% CI, 67.8 to 83.8) in patients without occult lymph node metastasis (*p* = 0.790) (Fig. 1 A; B).

A subgroup examination of the patients with occult lymph node metastasis due to elective ND (cN0/pN +) revealed a five-year OS rate of 72.9% (95% CI, 58.4 to 87.4). In contrast, patients with delayed isolated lymph node metastasis during the course of treatment (rT0rN +) exhibited an OS rate of 85.6 (95% CI, 76.0 to 95.2). There was no statistically significant difference when compared to patients without occult lymph node metastases (*p* = 0.613).

### Disease-free survival

In total, 167 events (38.1%) occurred. In patients without ND there were 137 events (41.6%) compared to 30 (27.5%) in the elective ND group. At five years, the corresponding DFS rates were 34.9% (95% CI, 25.2 to 44.6) and 58.1% (95% CI, 55.0 to 61.2) (p = 0.024).

In patients without occult lymph node metastasis there were 135 events (33.5%) and 18 (51.4%) further events after their diagnosis of occult lymph node metastasis. The rates of five-year DFS were 51.1 (95% CI, 42.9 to 59.3) and 42.0 (95% CI, 28.8 to 55.2) (p = 0.388). Without correction, the five-year DFS rate for patients who have developed occult lymph node metastasis since completing primary therapy was 5.3% (95% CI, 0.0 to 24.9) (p < 0.001) (Fig. 1 C; D). In the subgroup analysis for five-year DFS after detection of occult lymph node metastasis during elective ND (cN0/pN +) and for further recurrences in patients with occult lymph node metastases during the course of follow-up (rT0rN +), a five-year DFS of 31.3% (95% CI, 6.6 to 56.0 and 55.9 (95% CI, 49.5 to 62.3) was observed (p = 0.232).

### Subgroup analyses

**Fig. 2:**
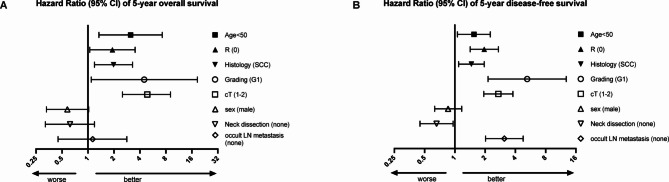
Hazard-ratio of five-year overall- (**A**) and disease-free survival (**B**) depending on different variables. [LN = lymph node; SCC = squamous cell carcinoma].

The five-year OS and DFS was also analyzed regarding different subgroups. In the univariate analysis, cT1-2, low-grade carcinoma, SCC, R0-resection and age below 50 years were identified as predictive factors for an improved five-year OS. In terms of DFS, the absence of occult lymph node metastases, performance of an elective neck dissection, cT1-2, low-grade carcinoma, SCC, a surgical R0 resection, and an age below 50 years was associated with superior survival. In the multivariate analysis, there were no independent predictive factors for five-year OS. For five-year DFS the multivariable analysis revealed the absence of occult lymph node metastasis [HR, 2.88 (95% CI, 1.60 to 5.18), p < 0.001], small tumor size (cT1-2) [HR, 1.59 (95% CI, 1.01 to 2.50), p = 0.046], low grade tumors (G1) [HR, 3.57, (95% CI, 1.09 to 11.71), p = 0.036], and R0-resection [HR, 1,76 (95% CI, 1.07 to 2.88), p = 0.025] to be predictive for a better five-year DFS (Fig. 2; Supplementary Table 1 [S]).

### Patterns of recurrence

The exact pattern of disease recurrence of patients without and elective ND is shown in Table 3. Most relapses were local in both neck therapy regimens. Isolated nodal recurrence occurred in 23 patients (7.0%) without ND and in five patients (4.6%) with elective ND. Locoregional recurrence was defined as simultaneous local and lymphonodal tumor manifestation and occurred almost equally in both groups. In both, locoregional and isolated nodal failure, pN1-status (n = 14; 34.1%) was most common, with the most frequent manifestation in levels I-II (n = 26; 63.4%). The localization was almost exclusively ipsilateral to the former primary, with a total of five bilateral lymph node recurrences.

**Table 3:** Patterns of recurrence*.*

*Recurrence*	*No Neck Dissection* *(n* = *164 of 329)* *(% of patients without initial ND)*	*Elective Neck Dissection* *(n* = *33 of 109)* *(% of patients with initial ND)*	*P*
Isolated local	64 (19.5%)	18 (16.5)	0.158
Isolated nodal	23 (7.0%)	5 (4.6%)	0.374
Isolated distant metastasis	27 (8.2%)	6 (5.5%)	0.132
Locoregional recurrence*	11 (3.3%)	2 (1.8%)	0.075
Combined metastasis**	39 (11.9%)	2 (1.8%)	**0.007**

*defined as local + *nodal recurrence.*

**defined as local/nodal/locoregional recurrence + *distant metastasis.*

Significant values are in [bold].

Neck dissection was performed on all of the 41 patients with lymphatic recurrence (both isolated nodal and locoregional), resulting in a mean number of dissected lymph nodes of 23.5 (95% CI, 17.6 to 29.3) with positive lymph nodes of 2.4 (95% CI, 1.7 to 3.1).

## Discussion

The incidence of occult lymph node metastasis in sinonasal malignancies is described as up to 16% and is highly depending on the histologic subtype^20,22^. For SCC of the sinonasal tract, the most recent systematic review reports a rate of 12.5% for histologically proven occult nodal metastasis, with nearly half of them being pathologically N2 disease^20^. Other authors report a rate of isolated nodal failure from 4.8 to 7.3% in patients with initial cN0 maxillary sinus carcinoma^11,12,23^. Sinonasal adenoid cystic carcinoma revealed an even higher rate of 16% (4/24) of occult lymph node metastasis in patients who underwent elective ND^24^. For other histologic subtypes the incidence is generally lower. In studies of sinonasal adenocarcinoma populations, the incidence of lymph node metastasis at diagnosis or during follow-up remains consistently low, typically under 10%^25–27^. These findings align with our own data. The overall rate of occult lymph node metastases was found to be 8.0%, with these metastases originating from SCC (10.3%), adenocarcinoma (4.2%), SNUC (10.5%) and neuroendocrine carcinoma (22.2%). Approximately a quarter of these patients exhibited lymph node metastasis undergoing an elective ND.

Histology proved to be a risk factor for the occurrence of occult metastasis. While NEC was identified as predictor for occult lymph node metastasis in univariate analysis, this association was not substantiated in multivariate analysis. This outcome is likely attributable to the limited number of cases and the concomitant high confidence interval. The only independent risk factor for the occurrence of occult lymph node metastases was therefore SCC histology compared to the other tumor entities in our cohort.

Furthermore, a safe resection margin has been identified as possible, but not significant, protective factor for the occurrence of occult lymph node metastases. However, none of these risk factors can be reliably assessed before radical tumor resection and removal of lymph node metastases. This results in a significant diminution of their clinical utility in evaluating preoperative lymph node status. To our knowledge, there have been no studies to date evaluating risk factors for the occurrence of occult lymph node metastases in sinonasal malignancies. However, multiple studies have shown that advanced primary tumor stage (T3/T4), SCC, high tumor grade, and specific anatomic subsites such as the maxillary sinus are risk factors for a nodal involvement at the time of diagnosis^16,20,22,28^. In other tumor entities the phenomenon of occult metastases has been adequately investigated. In the context of oral cavity carcinoma, rates ranging from 15 to 35% have been observed in cases of T1/T2N0 carcinomas^29–32^.

According to our theory, isolated lymph node recurrences in the absence of prior neck dissection are hypothesized to be attributable to occult lymph node metastases that were already present at the time of diagnosis. Tarsitano et al. demonstrated, based on mitochondrial DNA analysis, that delayed lymph node metastases are predominantly clonally derived from the primary tumor^33^. This observation further supports the hypothesis that such metastases represent the progression of previously undetected micrometastatic disease rather than the development of de novo metastatic lesions.

However, the phenomenon of delayed nodal metastasis is not yet investigated sufficiently in the context of sinonasal carcinomas. In oral cavity SCC, the occurrence of delayed metastases has been proven to be influenced by the location and extent of the primary tumor, including predictive factors as perineural invasion, poor differentiation grade, or lymphangiogenic marker expression (Flt-4)^34–36^. These findings demonstrate that tumor biology can also exert an additional influence on the occurrence of isolated regional lymph node recurrences.

Retropharyngeal lymph node involvement in sinonasal carcinoma is uncommon but associated with advanced disease and poor prognosis^37^. Clinical and imaging studies describe a retropharyngeal lymph node involvement in a subset of patients with sinonasal carcinoma, with reported incidences ranging from 15 to 30% depending on tumor location and histology^37–40^. Nevertheless, in our cohort, no cases of retropharyngeal lymph node involvement were observed.

A positive lymph node status is generally seen as a predictor of worse survival^41–43^. In our cohort, the presence of occult lymph node metastases showed no significant influence on OS, respectively. However, DFS was significantly reduced, but when adapted to secondary DFS after diagnosis of occult LN metastasis, patients showed similar DFS compared to the group without occult metastases. The study also demonstrated that patients who did not undergo ND exhibited a significantly poorer five-year DFS. While there were no significant differences in the location of tumor recurrences regarding the neck treatment, except for combined recurrences, there was a consistently higher risk of recurrence in patients without elective neck dissection at every recurrence site.

To date, there are no uniform guidelines or treatment recommendations regarding the performance of elective ND for sinonasal carcinoma. Besides the heterogeneity of the various tumor entities and their anatomical location, the existent literature on this issue is discordant. Sangal et al. stated that patients with T3 tumors of the maxillary sinus who underwent ND exhibited a superior DFS, although this was not observed in patients with T4 tumors^44^. Crawford et al. reported that there was no improvement in OS in patients with elective ND for cN0M0 sinonasal carcinoma^45^. In a recent meta-analysis elective ND or elective nodal irradiation were found to significantly reduce regional recurrence rates but have not demonstrated a survival benefit^17^.

The discrepancy between enhanced DFS yet unchanged OS with elective ND in sinonasal carcinoma is likely attributable to two converging factors: First, OS is driven by T-stage, local control and distant metastasis. A recent multicenter pooled analysis demonstrated that the strongest independent predictors of OS are advanced T-stage, histology, and nodal involvement at presentation, not whether the neck was electively treated^46^. Local recurrence and distant metastasis are the lethal events, and ND does not mitigate either of these. Second, salvage options for isolated regional recurrence may preserve OS. In the event of a regional recurrence in the absence of prior ND, treatment options may include surgery or radiation, thereby mitigating the potential survival disadvantage^47,48^. This effect dilutes the OS benefit of elective neck treatment.

Elective ND is generally recommended for carcinomas with a 15% rate of occult metastases^49^. In the present study, however, the rate was found to be 8.0%. The morbidity associated with elective ND is generally considered low to moderate, but it is not negligible. Anatomic structures in the vicinity, such as the marginal branch of the facial nerve, accessory nerve, hypoglossal nerve, vagus nerve, and lingualis nerve, have been observed to be susceptible to injury and potential morbidity during ND^50^. Alternatively, SLNB is emerging as a low-morbidity alternative for staging in cN0 disease, but its role remains investigational^51,52^. Many authors have demonstrated that SLNB is associated with significantly fewer postoperative complications, better shoulder function, shorter scar length, reduced hospital stay, and fewer adverse events, while maintaining equivalent oncologic outcomes in terms of OS and DFS in early oral cancer^53–55^.

This study is strengthened by a large sample size, multicenter data, and a long follow-up, including the management of lymph node recurrence. However, several limitations should be acknowledged. First, its retrospective design may limit generalizability and introduce potential selection and indication bias. Patients for whom elective ND was recommended were presumably those with higher risk features. Additionally, the heterogeneity of histological subtypes may introduce variability in metastatic behavior and treatment responses.

## Conclusion

This multicenter study with unprecedented depth of data demonstrated a comparatively low risk of occult lymph node metastases occurring in sinonasal malignancies. Nevertheless, disease-free survival is significantly reduced in these patients.

The overall low rate of occult metastases indicates that elective ND is not necessary and carries an additional risk of morbidity. In tumor entities with an increased risk of occult metastases, such as SCC, the potential benefits of elective neck dissection should be evaluated.

## Electronic supplementary material

Below is the link to the electronic supplementary material.

Supplementary file 1 (DOCX 15 kb)

## Data Availability

The datasets used and/or analyzed during the current study are available from the corresponding author on reasonable request.
