# Supplementary material for: A multicenter study on occult lymph node metastases in sinonasal malignancies
Source: Sci Rep. 2026 May 24;16:16025. doi: 10.1038/s41598-026-47890-z (PMC13199410; doi:10.1038/s41598-026-47890-z)
Supplement: Supplementary file 1 — (DOCX 15 kb) [file 41598_2026_47890_MOESM1_ESM.docx]

*SUPPLEMENTARY:*

*Table 1 [S].*

*Uni- and multivariate analysis for 5-year overall survival (A) and disease-free survival (B)*

| **A** |  | ***Univariate analysis*** | | | ***Multivariate analysis*** | | |
| --- | --- | --- | --- | --- | --- | --- | --- |
| **Subgroup** | **reference** | **HR** | **95% CI** | **p** | **HR** | **95% CI** | **p** |
| Occult LN metastasis | none | 1.132 | 0.45–2.82 | 0.791 |  |  |  |
| Neck dissection | none | 0.619 | 0.32–1.19 | 0.150 | 0.532 | 0.17–1.70 | 0.286 |
| Sex | male | 0.577 | 0.33–1.02 | 0.058 | 0.601 | 0.24–1.54 | 0.288 |
| cT | cT1-2 | **4.768** | **2.51–9.05** | **< 0.001** | 1.941 | 0.82–4.58 | 0.130 |
| Grading | G1 | **4.484** | **1.09–18.53** | **0.038** | 3.124 | 0.40-24.22 | 0.276 |
| Histology | SCC | **1.981** | **1.19–3.29** | **0.008** | 1.480 | 0.58–3.77 | 0.412 |
| R | R0 | **1.921** | **1.05–3.52** | **0.035** | 1.062 | 0.41–2.77 | 0.902 |
| Age | ≤50 | **3.116** | **1.34–7.25** | **0.008** | 3.157 | 0.74–13.45 | 0.120 |

| **B** |  | ***Univariate analysis*** | | | ***Multivariate analysis*** | | |
| --- | --- | --- | --- | --- | --- | --- | --- |
| **Subgroup** | **reference** | **HR** | **95%** | **p** | **HR** | **95% CI** | **p** |
| Occult LN metastasis | none | **3.106** | **2.02–4.78** | **< 0.001** | **2.875** | **1.60–5.18** | **< 0.001** |
| Neck dissection | none | **0.657** | **0.45–0.96** | **0.028** | 0.586 | 0.33–1.04 | 0.068 |
| Sex | male | 0.857 | 0.63–1.17 | 0.325 |  |  |  |
| cT | cT1-2 | **2.706** | **1.94–3.78** | **< 0.001** | **1.586** | **1.01–2.50** | **0.046** |
| Grading | G1 | **5.242** | **2.14–12.84** | **< 0.001** | **3.567** | **1.09–11.71** | **0.036** |
| Histology | SCC | **1.461** | **1.09–1.96** | **0.011** | 0.933 | 0.57–1.54 | 0.785 |
| R | R0 | **1.959** | **1.42–2.71** | **< 0.001** | **1.759** | **1.07–2.88** | **0.025** |
| Age | ≤50 | **1.549** | **1.06–2.25** | **0.022** | 1.755 | 0.98–3.16 | 0.061 |

*[LN = lymph node; SCC = squamous cell carcinoma]*
